# First record of *Myotis albescens* (Chiroptera, Vespertilionidae) in French Guiana

**DOI:** 10.3897/BDJ.3.e5314

**Published:** 2015-05-29

**Authors:** Ricardo Moratelli, Maël Dewynter, Marguerite Delaval, François Catzeflis, Manuel Ruedi

**Affiliations:** ‡ Fiocruz Mata Atlântica, Fundação Oswaldo Cruz, CEP 22713-375, Rio de Janeiro, Brazil; § Groupe Chiroptères de Guyane, 15 cité Massel, F-97300, Cayenne, French Guyana; | Institut des Sciences de l’Evolution, CNRS UMR-5554, Université Montpellier-2, F-34095, Montpellier, France; ¶ Department of Mammalogy and Ornithology, Natural History Museum of Geneva, BP 6434, 1211, Geneva 6, Switzerland

**Keywords:** Geographic distribution, Guiana Shield, *
Myotis
*, Myotinae, South America

## Abstract

**Background:**

*Myotis albescens* occurs from Mexico southward to Uruguay and Argentina. The species is known for all South American countries except French Guiana and Chile.

**New information:**

Based on one specimen recently collected in French Guiana we fill part of the gap in the distribution of the species in South America. *Myotis albescens* occurs in the Guiana Shield with other four congeners, from which it can be distinguished by external and skull traits. As an aid to future identifications, we provide a key to this assemblage.

## Introduction

*Myotis albescens* was described by Etienne Geoffroy-Saint-Hilaire (1806) based on the Azara’s *chauve-souris douzième* (or *chauve-souris brune-obscure*) from Paraguay (Azara 1801). The original material was not preserved (Wilson 2008), and the type locality was fixed by neotype designation in Yaguaron, Paraguarí, Paraguay by LaVal (1973).

The species has a continental distribution, occurring in different habitats from southern Veracruz, Mexico, southward through Central America to Uruguay and Argentina on the east side of the Andes, and to Ecuador on the west side of the Cordillera (Wilson 2008, Braun et al. 2009). Analyses of morphological and morphometric variation for South American populations revealed a gradient in the size of skull, with larger specimens in the south, but with populations showing a cohesive pattern for size and shape of skull throughout the continent. Beyond the geographical component, intrapopulational variation was observed for skull size and shape, and pelage colour (Moratelli and Oliveira 2011).

There are records of *M. albescens* supported by vouchers for all South American countries except French Guiana and Chile (Wilson 2008, Moratelli et al. 2013). Due to the apparent absence of natural barriers and high plasticity, the occurrence of *M. albescens* in French Guiana is expected. However, Simmons and Voss (1998) did not find evidence of the species in the country. On the other hand, Charles-Dominique et al. (2001) listed *M. albescens* for French Guiana without informing the source of the record. This was followed by Lim et al. (2005), who included *M. albescens* in the checklist of bats from French Guiana. However, Catzeflis et al. (2013) suggested removing this species from the list of the country until new evidence supported by vouchered material could prove its occurrence. Nogueira et al. (2014) used the same criterion—presence of vouchers in collections—for building their checklist of Brazilian bats. We agree with this position because vouchers are the primary verifiable and testable evidence of a species existence and occurrence, and they are particularly important for small mammals due to difficulties in identifying individuals under field conditions (Moratelli 2014). Based in one specimen deposited in the Muséum d'histoire naturelle, Geneva, Switzerland (MHNG 1990.017), we confirm the occurrence of *M. albescens* in French Guiana. Along with this distribution extension, we provide a key to the assemblage of *Myotis* from the Guiana Shield.

## Materials and methods

The MHNG 1990.017 is an adult female (weight 5.5 grams; forearm length 35.8 millimetres) collected by M. Dewynter (original field number V-2984) at Rivière des Cascades, municipality of Montsinery, about 27 km SW of Cayenne, French Guiana (04°45'01" N, 52°29'08" W), on 31 July 2011 at an elevation of 42 m. The specimen was caught by hand, inside a hollow log. It is preserved in spirit, with skull prepared separately. The basicranium is broken, which prevented us from taking several measurements, but the rostrum and braincase—structures useful in identifications—are intact.

This specimen was directly or indirectly compared with more than 3,800 vouchers of New World *Myotis*, representing all South and Central American species currently recognized, and some others pending formal description as well. Among the material examined are 750 representatives of *M. albescens*, including the neotype (American Museum of Natural History [AMNH] 205195) from Paraguarí, Paraguay. This set of specimens covers the entire distribution range of the species, and comprises most (if not all) of the morphological variation currently known for *M. albescens*.

Measurements are reported in millimetres (mm) and are from adults only. Dimensions were taken using digital callipers accurate to 0.02 mm, and craniodental measurements were taken under binocular microscopes with low magnification (usually 6x). Measurements include forearm length (FA), third metacarpal length (3MC), braincase breadth (BCB), interorbital breadth (IOB), postorbital breadth (POB), breadth across canines (BAC), breadth across molars (BAM), maxillary toothrow length (MTL), length of the upper molars (M1M3), and mandibular length (MAL). These measurements are defined in Moratelli et al. (2013). Values were rounded off to 0.1 mm throughout the text because this is the smallest unit that allows accurate repeatability with calipers (Voss et al. 2013).

## Taxon treatments

### Myotis albescens

(Étienne Geoffroy St.-Hilaire, 1806)

#### Materials

- **Type status:** Other material. **Occurrence:** catalogNumber: MHNG 1990.017; occurrenceRemarks: caught by hand, inside a hollow log; sex: female; lifeStage: adult; preparations: Ethanol 70o GL; disposition: in collection; **Taxon:** scientificName: Myotis albescens; kingdom: Animalia; phylum: Chordata; class: Mammalia; order: Chiroptera; family: Vespertilionidae; genus: Myotis; specificEpithet: albescens; taxonRank: species; scientificNameAuthorship: (É. Geoffroy-St.-Hilaire, 1806); vernacularName: Silver-tipped myotis; nomenclaturalCode: ICZN; taxonomicStatus: accepted; **Location:** higherGeography: French Guiana; Montsinery; Rivière des Cascades; continent: South America; country: French Guiana; stateProvince: Cayenne; municipality: Montsinery; locality: Cayenne, 27 km SW; verbatimElevation: 42 m; verbatimCoordinates: 04°45'01"N 52°29'08"W; verbatimLatitude: 04°45'01"N; verbatimLongitude: 52°29'08"W; verbatimCoordinateSystem: degrees decimal minutes; **Event:** eventDate: 2011-07-31

#### Diagnosis

Presence of a fringe of hairs along the trailing edge of uropatagium, long and silky pelage with frosted appearance on the dorsum, ears 9–14 mm in length, broad interorbital and postorbital constrictions, and globular braincase.

#### Distribution

Southern Veracruz, Mexico, southward through Central America to Uruguay and Argentina.

## Identification Keys

### Key to species of Myotis from the Guiana Shield

**Table:** 

1	Fringe of hairs along the trailing edge of uropatagium present	*M. albescens*
–	Fringe of hairs along the trailing edge of uropatagium absent	2
2	Dorsal fur woolly	3
–	Dorsal fur silky	4
3	Fur on dorsum of uropatagium extending along tibia to foot; fur on plagiopatagium along body distributed from elbow to tibia	*M. keaysi*
–	Fur on dorsum of uropatagium reaching knee or below; fur on plagiopatagium along body either absent or extremely sparse	*M. riparius*
4	Frontals steeply sloping; parietals rounded posteriorly in lateral view; greatest length of skull, including incisors, 14.4 mm or more	*M. oxyotus*
–	Angle of slope of frontals variable; parietals flattened and dorsal profile inclined forward; greatest length of skull, including incisors, less than 14.0 mm	*M. nigricans*

French Guiana is part of the Guiana Shield that also comprises Guyana, Suriname, the Venezuelan regions of Amazonas, Bolívar, and Delta Amacuro (see Lim et al. 2005:81, fig. 7), as well as the Brazilian states of Pará (part), Amapá, Amazonas, and Roraima. Along with *M. albescens*, four other species of *Myotis* are currently recognised for this region (see Lim et al. 2005): *M. nigricans* (Schinz, 1821), *M. oxyotus* (W. Peters, 1866), *M. keaysi* J. A. Allen, 1914, and *M. riparius* Handley, 1960. Among them, *M. nigricans* and *M. riparius* also occur in French Guiana (Simmons and Voss 1998). As an aid to future identifications, we provide a key to this assemblage.

## Analysis

Like other Neotropical *Myotis*, there is no one single character distinguishing *M. albescens* from its congeners. However, the species can be easily identified based on the following set of traits: presence of a fringe of hairs along the trailing edge of uropatagium, long and silky pelage with frosted appearance on the dorsum, ears 9–14 mm in length, broad interorbital and postorbital constrictions, and globular braincase. Although this set of traits is not fully present in some specimens, the presence of a fringe of hairs along the trailing edge of the uropatagium has been recorded in 99.4% of the specimens examined (500 out of 503) throughout the entire distribution range of the species, and is useful to distinguish it from all Neotropical species but *M. atacamensis* (Lataste, 1892) and *M. levis* (I. Geoffroy, 1824). *Myotis atacamensis* occurs from western Peru to northern Chile (Wilson 2008), and is consistently paler and smaller than *M. albescens*. *Myotis levis* occurs from the mountains of South-eastern Brazil southward to Uruguay (Wilson 2008​). It may resemble *M. albescens* in the pelage colour but can be easily distinguished by larger skull, and longer ears and fur.

The MHNG 1990.017 has a distinctive fringe of hairs along the trailing edge of uropatagium, the rostrum is short, and the braincase is globular. Additionally, all measurements fit with those of *M. albescens* from other localities in the north of South America (Table 1). Thus, the MHNG 1990.017 perfectly fits in the description of *M. albescens*, and it is recognized as such here.

## Discussion

With this record, *M. albescens* is confirmed (by vouchered material) for French Guiana and for all South American countries except Chile, from which the species is possibly absent. Its southernmost record on the west side of the Andes is in the semideciduous forests of north-western Peru (see Wilson 2008:471, map 272), a region in the Ecuadorian Province, extreme south of the Pacific Dominion (see Morrone 2014:24). One of us (RM) examined collections from the South American Transition Zone, particularly those from the Desert, Puna and Atacaman provinces (that together cover the north of Chile), and there is no evidence of *M. albescens* in the region. Geographically, the records closest from Chile are on the east side of the Andes, in the Chacoan Province of northern Argentina (LaVal 1973, Wilson 2008). The current known distribution for *M. albescens* in South America seems to be a good approximation of the real distribution of the species in the continent. Other putative occurrences of *M. albescens* outside this well-documented distribution are based on poorly documented (or unverifiable) field identification that are likely erroneous. This stresses the importance of vouchered and carefully identified specimens to appropriately record species occurrences in such difficult taxonomic groups as the Neotropical *Myotis* (Moratelli 2014).

Although this record does not add any biological information for the species, it is useful to more accurately describe the spectacular biodiversity of French Guiana, and emphasizes its rich biota, which is taxonomically and ecologically highly diversified for bats. Paracou in French Guiana concentrates one of the highest species diversity in the world, with ca. 78 species (see Simmons and Voss 1998). About 150 bat species are currently known for the Guiana Shield, 104 (including *M. albescens*) of which occur in French Guiana (Lim et al. 2005, Catzeflis et al. 2013).

## Supplementary Material

XML Treatment for Myotis albescens

## Figures and Tables

**Table 1.** Measurements (in mm) of the MHNG 1990.017 from French Guiana, and descriptive statistics for samples of *Myotis albescens* from northern South America. N = sample size, including adults only, and with sexes combined. See Methods for variable abbreviations.

	**French Guiana**	**Amazon**	**Venezuela**	**Peru**
	**MHNG 1990.017**	**Mean (Range) N**	**Mean (Range) N**	**Mean (Range) N**
FA	35.6	34.6 (32.2–36.7) 80	–	35.8 (34.4–37.2) 9
3MC	32.9	32.3 (30.3–34.5) 81	–	32.9 (31.5–34.7) 17
BCB	6.9	6.7 (6.4–7.0) 66	6.8 (6.5–7.0) 11	6.8 (6.5–7.0) 22
IOB	4.5	4.5 (4.2–4.8) 67	4.5 (4.4–4.7) 11	4.6 (4.0–4.8) 23
POB	3.8	3.8 (3.5–4.1) 67	3.7 (3.6–3.9) 11	3.8 (3.6–4.0) 23
BAC	3.7	3.6 (3.3–3. 8) 65	3.6 (3.5–3.8) 11	3.7 (3.4–5.4) 20
BAM	5.3	5.3 (4.9–5.6) 67	5.5 (5.2–4.8) 11	5.4 (5.3–5.7) 22
MTL	5.1	4.9 (4.7–5.3) 67	5.0 (4.9–5.1) 11	5.1 (4.8–5.2) 23
M1M3	2.8	2.7 (2.6–2.9) 67	2.9 (2.8–2.9) 10	2.9 (2.8–3.0) 23
MAL	9.9	9.6 (9.3–10.2) 18	9.8 (9.7–10.1) 6	9.9 (9.6–10.2) 9
